# Pulmonary endothelial activation caused by extracellular histones contributes to neutrophil activation in acute respiratory distress syndrome

**DOI:** 10.1186/s12931-016-0472-y

**Published:** 2016-11-21

**Authors:** Yanlin Zhang, Li Guan, Jie Yu, Zanmei Zhao, Lijun Mao, Shuqiang Li, Jinyuan Zhao

**Affiliations:** Research Center of Occupational Medicine, Third Hospital of Peking University, No.49 North Garden Road, Haidian District, Beijing, 100191 People’s Republic of China

**Keywords:** Extracellular histones, Acute respiratory distress syndrome, Inflammation, Endothelium, Neutrophil

## Abstract

**Background:**

During the acute respiratory distress syndrome (ARDS), neutrophils play a central role in the pathogenesis, and their activation requires interaction with the endothelium. Extracellular histones have been recognized as pivotal inflammatory mediators. This study was to investigate the role of pulmonary endothelial activation during the extracellular histone-induced inflammatory response in ARDS.

**Methods:**

ARDS was induced in male C57BL/6 mice by intravenous injection with lipopolysaccharide (LPS) or exogenous histones. Concurrent with LPS administration, anti-histone H4 antibody (anti-H4) or non-specific IgG was administered to study the role of extracellular histones. The circulating von Willebrand factor (vWF) and soluble thrombomodulin (sTM) were measured with ELISA kits at the preset time points. Myeloperoxidase (MPO) activity in lung tissue was measured with a MPO detection kit. The translocation of P-selectin and neutrophil infiltration were measured by immunohistochemical detection. For in vitro studies, histone H4 in the supernatant of mouse lung vascular endothelial cells (MLVECs) was measured by Western blot. The binding of extracellular histones with endothelial membrane was examined by confocal laser microscopy. Endothelial P-selectin translocation was measured by cell surface ELISA. Adhesion of neutrophils to MLVECs was assessed with a color video digital camera.

**Results:**

The results showed that during LPS-induced ARDS extracellular histones caused endothelial and neutrophil activation, as seen by P-selectin translocation, release of vWF, an increase of circulating sTM, lung neutrophil infiltration and increased MPO activity. Extracellular histones directly bound and activated MLVECs in a dose-dependent manner. On the contrary, the direct stimulatory effect of exogenous histones on neutrophils was very limited, as measured by neutrophil adhesion and MPO activity. With the contribution of activated endothelium, extracellular histones could effectively activating neutrophils. Both inhibiting the endothelial activation with an anti-toll like receptor (TLR) antibody and inhibiting the interaction of the endothelium with neutrophil using an anti-P-selectin antibody decreased the degree of neutrophil activation.

**Conclusions:**

Extracellular histones are pro-inflammatory mediators in LPS-induced ARDS in mice. In addition to direct action to neutrophils, extracellular histones promote neutrophil adhesion and subsequent activation by first activating the pulmonary endothelium via TLR signaling. Thus, endothelial activation is important for extracellular histone-induced inflammatory injury.

## Background

ARDS is the leading cause of death in intensive care units with a mortality rate of at least 40% [1, 2]. Furthermore, the severity of ARDS is strongly associated with the incidence of multiple organ failure (MOF) [3]. The high mortality indicates that the key mechanism of the pathogenesis is not very clear. Sepsis is the most common risk factor for ARDS, which may represent one third of the cases [4, 5].

Uncontrolled inflammation is closely involved with the pathogenesis of ARDS [6]. Recently extracellular histones have been recognized as pivotal mediators of lethal systemic inflammatory diseases, both infectious and noninfectious [7–9]. More importantly, Freeman et al. have proved that extracellular histones can bind pulmonary capillary endothelial cells with priority through a charge-dependent interaction [10].

Neutrophil activation is viewed as a central event during the inflammatory response of ARDS, and consists of the recruitment of neutrophil to the pulmonary vasculature, adhesion to the lung endothelium and eventual activation [11, 12]. Yet the mechanism by which extracellular histones activate neutrophil is not clear. The adhesion of neutrophil on the pulmonary microvasculature requires P-selectin, an adhesion molecule constitutively stored within the Weibel-Palade bodies (WPBs) [13]. Upon endothelial activation, P-selectin can rapidly translocate to the cell surface and interact with P-selectin-glycoproteinligand-1 (PSGL-1) which is expressed on neutrophils. At the same time, vWF is released through exocytosis from WPBs, which is another critical contributor to the ongoing lung injury [14, 15]. Collectively, the lung endothelium plays a primary role by providing the surface platform for activating inflammation and coagulation cascade.

We hypothesized that activation of the pulmonary endothelium by extracellular histones may be a key step for neutrophil activation in the pathogenesis of ARDS. Rationally, a better understanding of the pathogenic mechanism may facilitate effective therapies.

## Methods

### Reagents

Calf thymus histones (CTH) were purchased from Sigma (Dorset, UK). Goat antibodies to histone H4 were purchased from Cell Signaling Technology (MA, USA). The blocking antibodies against TLR2 (clone TL2.1), TLR4 (HTA125) and P-selectin (clone AK-4) were purchased from eBioscience (CA, USA). Antibodies for P-selectin, Ly6G and CD31 (PECAM-1) were purchased from Abcam (Cambridge, UK). A Cell Death Detection ELISA^PLUS^ was purchased from Roche Diagnostics (IN, USA). ELISA kits for vWF and sTM were purchased from Cusabio Biotech (Wuhan, China).

### Animal studies

Six- to eight-week-old male C57BL/6 mice, weighting 18–20 g, were purchased from the Experimental Animal Center of Peking University (Peking, China). They were housed in an air-conditioned room at 25 °C with a 12 h dark–light cycle and allowed to acclimate upon arrival for 3 days before experimentation. All experimental protocols of this study were approved by the Institutional Animal Care and Use Committee of Health Sciences Center, Peking University (protocol no. LA201284). All the procedures strictly followed the institutional and federal guidelines. The efforts were made to minimize animal suffering. When the experiment was completed, the mice were humanely sacrificed by injecting ketamine (100 mg/kg) and xylazine (8 mg/kg) via tail vein before cervical dislocation.

### Preparation and injection of anti-histone H4 antibody

Mouse anti-H4 antibody was prepared following the previously described protocol [16]. Nonspecific mouse IgG was used as the control. The anti-H4 antibody (20 mg/kg) and the nonspecific IgG (20 mg/kg) were injected once via the tail vein 30 min prior to LPS infusion.

### LPS or exogenous histones induced ARDS

ARDS was induced by intravenous injection of LPS (2–10 mg/kg, 24 h) or CTH (40 mg/kg, 6 h). LPS and CTH were diluted with 0.9% (w/v) normal saline. The control mice underwent the same procedure with intravenous injection of equivalent normal saline.

### Western blot of cell supernatant

Protein concentration was determined with the Bicinchoninic Acid Protein Assay Kit (Sigma, MO, USA). Equal amount of proteins (100 μg) were subjected to electrophoresis using 12% (w/v) SDS-polyacrylamide gels. Separated proteins were transferred onto nitrocellulose membranes. The membranes were then incubated with anti histone H4 antibody.

### Immunohistochemical detection of P-selectin

Immunohistochemical localization of P-selectin in lung sections was determined according to the protocol [17]. Quantification of P-selectin positive venules was accomplished with the protocol [18]. Fifty venules were analyzed per tissue section, 24 sections were examined per group, and the percentage of positive-staining venules was calculated.

### MLVEC isolation and characterization

MLVECs were isolated by using the method [19]. Briefly, peripheral lung tissue was diced into a size about 1 mm^3^ and cultured in a 60-mm culture dish. The adherent cells were purified with biotin-conjugated rat anti-mouse CD31 antibody and cultured in endothelial growth medium-2 (EGM-2) supplemented with 10% FBS. The MLVECs were characterized by their cobblestone morphology and staining for factor VIII-related antigen (Sigma, MO, USA).

### Immunofluorescence confocal laser microscopy

MLVECs were challenged with LPS for 6 h in 6-well cell culture plate coated with poly-L-lysine. Then the supernatant was transferred into another cell culture plate with unchallenged MLVECs. After 10 min of incubation the cells were fixed with formalin. An anti-H4 primary antibody and a FITC-labeled secondary antibody were used to visualize extracellular histone H4 and DAPI (Vector, CA, USA) was used to stain the nuclei. Immunofluorescence assay was performed under a confocal laser microscope (Leica, Germany).

### Cell surface ELISA

Endothelial P-selectin translocation was measured by cell surface ELISA as described previously [20]. MLVECs were challenged with CTH in the presence or absence of specific blocking antibody for TLR2 or TLR4. Reactions were stopped by removing stimulation medium and adding 1% paraformaldehyde for 20 min. The MLVECs were incubated with blocking solution (5% BSA) for 15 min, then with an anti-P-selectin antibody (1:100 dilution, 90 min). Peroxidase activity was quantified at 450 nm using a plate reader. The basal level of cell surface P-selectin from control MLVECs was normalized to 100%. Nonspecific binding was assessed by substituting primary antibodies with normal rabbit serum (Santa Cruz, CA, USA).

### Isolation of mouse neutrophils from bone marrow

Purification of bone marrow–derived neutrophils was performed according to a previously described protocol [21]. Bone marrow cells were flushed out by a syringe filled with Hanks’ balanced salt solution (HBSS, without calcium and magnesium) containing 2 mM EDTA. After red blood cells were removed the remaining cells were layered by Histopaque (Sigma-Aldrich, MO, USA) density gradient (3 ml Histopaque 1119 as bottom layer, 3 ml Histopaque 1077 as middle layer and 1 ml cell-containing PBS as upper layer). After centrifugation (2000 rpm for 40 min) the cells at the interface of Histopaque 1119 and Histopaque 1077 were collected. The purity of neutrophils was analyzed by Wright-Giemsa staining and viability was assessed by trypan blue dye exclusion assay.

### Neutrophil adhesion assay

Isolated neutrophils (1x10^5^ in 500 μl of 2% BSA-RPMI-1640) were added to MLVECs and incubated for 10 min. Adhesion of neutrophils to MLVECs was assessed with a color video digital camera adapted to a binocular microscope (Olympus, Japan). For each well, 3 fields of view were randomly selected and the neutrophils were counted and recorded as the number of adhered neutrophils/mm^2^. For the slight variations of basal neutrophil adhesion between experiments, the neutrophil adhesion was reported as a value relative to that of control (designated 100%) [22]. The neutrophil adhesion assay was done in three different conditions: (1) Neutrophils were challenged by CTH, then exposed to unchallenged MLVECs. (2) MLVECs were challenged by CTH, then exposed to unchallenged neutrophils. (3) Both MLVECs and neutrophils were challenged by CTH, then exposed to each other.

### Statistical analysis

The results are presented as mean ± SD. Statistical significance of differences among groups was determined by ANOVA followed by the Student-Newman-Keuls test. All statistical analyses were calculated using GraphPad Prism v5 (San Diego, CA, USA). *P* values of less than 0.05 were considered statistically significant.

## Results

### Role of extracellular histones in endothelial and neutrophil activation in LPS-induced ARDS

**Fig. 1:**
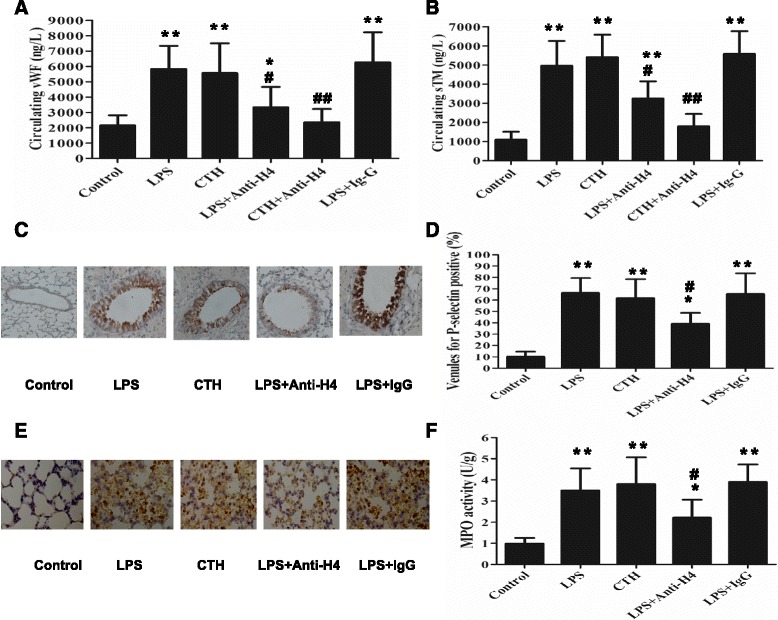
Role of extracellular histones in endothelial and neutrophil activation in mice with ARDS. Mice were challenged with intravenous LPS (10 mg/kg, 24 h) or CTH (40 mg/kg, 6 h). Anti-H4 antibody (20 mg/kg) or non-specific mouse IgG (20 mg/kg) was injected intravenously once 30 min prior to LPS injection. The levels of circulating vWF and sTM were measured by ELISA (**a**, **b**). The translocation of P-selectin was measured by immunohistochemical detection (**c**, **d**). Neutrophil infiltration in the lungs was confirmed by immunohistochemical analysis of the specific marker Ly6G and neutrophil activation was examined by MPO activity (**e**, **f**). Data are presented as mean ± SD (*n* = 6). The immunohistochemical results are representative of three similar experiments. *p < 0.05 vs. the control group, ** *p* < 0.01 vs. the control group; ^#^ *p* < 0.05 vs. the LPS group, ^##^ *p* < 0.01 vs. the LPS group

After intravenous injection of LPS, circulating vWF and sTM were elevated at 24 h. Similarly with LPS injection, mere CTH infusion also increased circulating vWF and sTM. Pre-treatment with an anti-H4 antibody attenuated the increase of circulating vWF and sTM, whereas non-specific IgG showed little effect (Fig. 1a, b).

The percentage of venules stained positively for P-selectin in pulmonary sections from control mice was very low (11 ± 2%). In contrast, infusion of LPS for 24 h resulted in a significant P-selectin translocation, which was shown as an increased percentage of venules stained positively for P-selectin (62 ± 9%, *P* < 0.01 versus the control). Additionally, infusion of CTH also caused an obvious P-selectin translocation. Pre-treatment with the anti-H4 antibody attenuated P-selectin translocation (Fig. 1c, d).

After LPS infusion for 24 h, neutrophil infiltration in the lung tissue was more prominent in comparison to the control group, which was indicated by the staining of the specific surface marker Ly6G (Fig. 1e). MPO activity in the lung tissue was also increased in LPS challenged mice (Fig. 1f). Infusion of CTH caused a similar increase in neutrophil infiltration and activation. Pre-treatment with the anti-H4 antibody attenuated the staining of Ly6G and MPO activity in the lungs.

### Effect of extracellular histones on endothelial activation in vitro

**Fig. 2:**
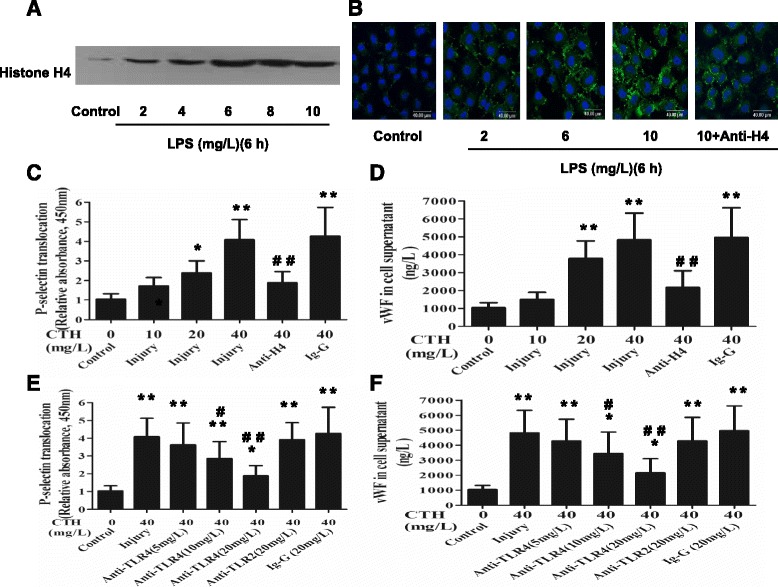
Effect of extracellular histones on endothelial activation in vitro. The MLVECs were challenged with LPS and then histone H4 in supernatant was measured by Western blot (**a**). After 10 min of incubation the binding of extracellular histones to the unchallenged endothelial cell membrane was examined by confocal laser microscopy (**b**). The MLVECs were treated with extracellular histones (1 h) and then P-selectin on endothelium was quantified by cell surface ELISA (**c**). The vWF in the supernatant was measured by ELISA (**d**). MLVECs were exposed to CTH and treated concurrently with anti-TLR2 or anti-TLR4 antibody. The inhibitory effect on endothelial activation was measured by P-selectin translocation (**e**) and release of vWF (**f**). Data are presented as mean ± SD (*n* = 6). The results of Western blot and confocal laser microscopy are representative of three similar experiments. *p < 0.05 vs. the control group, ** *p* < 0.01 vs. the control group; ^#^ *p* < 0.05 vs. the histones (40 mg/L) group, ^##^ *p* < 0.01 vs. the histones (40 mg/L) group

The extracellular histone H4 was nearly undetectable in the cell supernatant from the control MLVECs. After administration of LPS (2, 4, 6, 8, 10 mg/L, 6 h), histone H4 in the supernatant was increased in a dose dependent manner due to release of H4 from LPS-damaged MLVECs (Fig. 2a).

After MLVECs were incubated with various concentrations of LPS (2, 6, 10 mg/L, 6 h), the conditioned media was added to unchallenged cells. The extracellular histones in the supernatant notably accumulated on the plasma membrane of endothelium, and evidently outlined the endothelial membranes. The binding of H4 to the endothelial cell surface could be blocked by concurrent administration of the anti-H4 antibody (Fig. 2b).

Incubation with CTH (10, 20, 40 mg/L, 1 h) caused obvious P-selectin translocation and vWF release in a concentration dependent manner. Both P-selectin on the endothelial cell surface (*r* = 0.9976, *p* = 0.0024) and vWF in the supernatant (*r* = 0.9852, *p* = 0.0148) were significantly correlated with the concentrations of extracellular histones. Concurrent administration of the anti-H4 antibody significantly reduced the toxicity of extracellular histones (Fig. 2c, d).

In contrast to MLVECs that were challenged by histones alone, concurrent administration of an anti-TLR4 antibody markedly reduced P-selectin translocation (18 to 63% decrease versus the injury group) in a dose dependent manner (5–20 mg/L). An anti-TLR2 antibody only slightly reduced the P-selectin translocation (7% decrease versus the injury group) (20 mg/L) (Fig. 2e). Also, the anti-TLR4 antibody significantly reduced vWF release from the endothelium (21 to 54% decrease versus the injury group) in a dose dependent manner (5–20 mg/L). The anti-TLR2 antibody slightly reduced the vWF release (6% decrease versus the injury group) (20 mg/L) (Fig. 2f).

### Effect of histone-induced endothelial activation on neutrophil activation in vitro

**Fig. 3:**
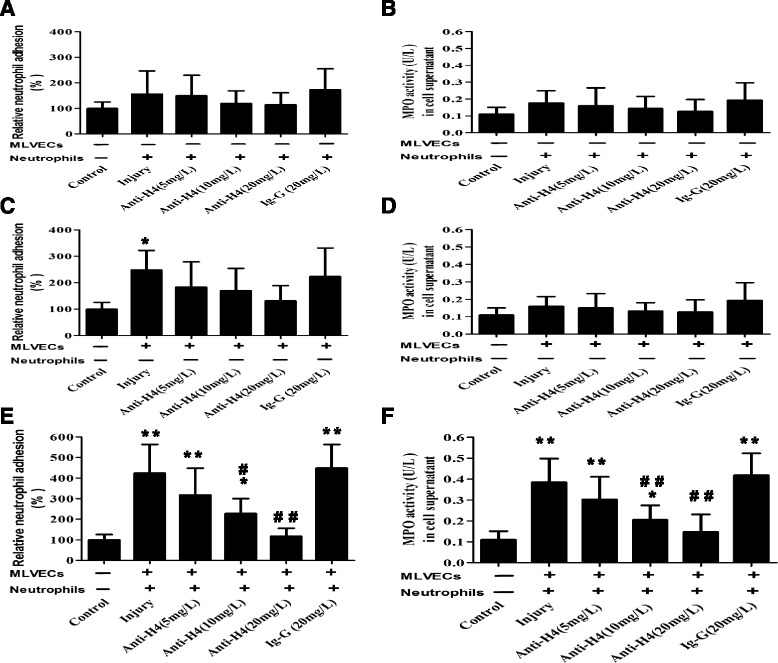
Effect of histone-induced endothelial activation on neutrophil activation. Neutrophils were challenged by CTH (40 mg/L, 1 h), then exposed to unchallenged MLVECs. The anti-H4 antibody or non-specific IgG was given concurrently with CTH. The relative percentage of neutrophil adhesion to MLVECs was determined by a cell surface adhesion assay under static conditions (**a**) and the MPO activity in the supernatant was measured by ELISA (**b**). MLVECs were challenged by CTH (40 mg/L, 1 h), then exposed to unchallenged Neutrophils. The relative percentage of neutrophil adhesion to MLVECs (**c**) and the MPO activity in the supernatant (**d**) were measured. Both MLVECs and neutrophils were challenged by CTH (40 mg/L, 1 h), then exposed to each other. The relative percentage of neutrophil adhesion to MLVECs (**e**) and the MPO activity in the supernatant (**f**) were measured. Data are presented as mean ± SD (*n* = 6). *P < 0.05 vs. the control group, **P < 0.01 vs. the control group; ^#^ *p* < 0.05 vs. the injury group, ^##^ *p* < 0.01 vs. the injury group

When neutrophils were challenged by CTH and exposed to unchallenged MLVECs, the percentage of neutrophil adhesion to MLVECs increased slightly (42% increase versus the control, *p* = 0.37) (Fig. 3a). MPO activity was also slightly elevated in comparison to control (48% increase versus the control, *p* = 0.26) (Fig. 3b).

When MLVECs were challenged by CTH and exposed to unchallenged neutrophils, the percentage of neutrophil adhesion to MLVECs was increased obviously (126% increase versus the control, *p* = 0.03) (Fig. 3c). But the effect on MPO activity was not evident (23% increase versus the control, *p* = 0.78) (Fig. 3d).

When both MLVECs and neutrophils were challenged by CTH, the percentage of adherent neutrophils increased sharply (318% increase versus the control, *p* = 0.005) (Fig. 3e). Additionally, MPO activity was also increased in comparison to the control (294% increase versus the control, *p* = 0.004) (Fig. 3f). Concurrent administration of the anti-H4 antibody (5–20 mg/L) with CTH (40 mg/L, 1 h) resulted in the inhibition of neutrophil adhesion and MPO activity.

### TLR signaling and P-selectin involved with endothelium-mediated neutrophil activation

**Fig. 4:**
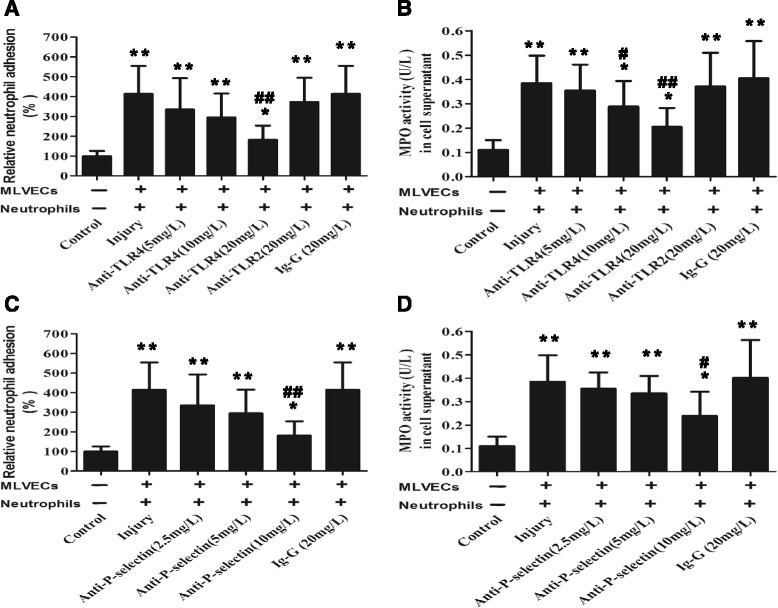
Roles of TLR signaling and P-selectin in endothelium-mediated neutrophil activation. MLVECs and neutrophils were challenged by CTH (40 mg/L, 1 h) and treated concurrently with anti-TLR2, anti-TLR4, anti-P-selectin antibody or non-specific IgG. The relative percentage of neutrophil adhesion to MLVECs (**a**, **c**) and the MPO activity in the cell supernatant (**b**, **d**) were measured. Data are mean ± SD of at least 6 experiments. *P < 0.05 vs. the control group, **P < 0.01 vs. the control group; ^#^ *p* < 0.05 vs. the injury group, ^##^ *p* < 0.01 vs. the injury group

In comparison to MLVECs and neutrophis that were challenged by histones alone, concurrent administration of an anti-TLR4 antibody markedly reduced the relative neutrophil adhesion (18 to 63% decrease versus the injury group) in a dose dependent manner (5–20 mg/L). An anti-TLR2 antibody only slightly reduced the relative neutrophil adhesion (7% decrease versus the injury group) (20 mg/L) (Fig. 4a).

Also, the anti-TLR4 antibody obviously reduced neutrophil MPO activity (9 to 54% decrease versus the injury group) in a dose dependent manner (5–20 mg/L). The anti-TLR2 antibody slightly reduced neutrophil MPO activity (6% decrease versus the injury group) (20 mg/L) (Fig. 4b).

Additionally, concurrent administration of an anti-P-selectin antibody markedly reduced the relative neutrophil adhesion (21 to 62% decrease versus the injury group) in a dose dependent manner (2.5–10 mg/L) (Fig. 4c).

The anti-P-selectin antibody also obviously reduced neutrophil MPO activity (12 to 37% decrease versus the injury group) in a dose dependent manner (2.5–10 mg/L) (Fig. 4d).

## Discussion

While ARDS can be caused by diverse underlying diseases, the uncontrolled overwhelming inflammatory response to the initial stimuli is viewed as a hallmark of ARDS. Damage-associated molecular patterns (DAMPs) are considered to be a major pathway of uncontrolled inflammation in addition to the classic pathogen-associated molecular patterns (PAMPs) [23].

In this study, we showed that both endothelial and neutrophil activation were evident in LPS-induced ARDS in mice, as measured by P-selectin translocation, release of vWF from WPBs, an increase of circulating sTM, lung neutrophil infiltration and increased MPO activity. In vitro experiments showed that extracellular histones directly bound and activated MLVECs in a dose-dependent manner, increasing P-selectin translocation and release of vWF from WPBs. Additionally, TLRs were closely involved with extracellular histone-induced endothelial activation as concurrent administration with selective TLR4 or TLR2 inhibitor inhibited P-selectin translocation and vWF release in a dose dependent manner.

In contrast, the direct stimulatory effect of CTH on neutrophils was very limited, displayed as modest increase in neutrophil adhesion and MPO activity. However, in the presence of activated endothelial cells, CTH effectively activated neutrophils. This suggests that by first activating the pulmonary endothelium, extracellular histones can then induce neutrophil adhesion and subsequent activation as is observed in the pathogenesis of ARDS. Both inhibiting endothelial activation with an anti-TLR antibody, and inhibiting the interaction of endothelial cells and neutrophils with an anti-P-selectin antibody, decreased the degree of neutrophil activation. These findings indicate that endothelial activation is essential for extracellular histone-induced inflammatory injury.

In support of these findings, Westman et al. has also showed that during neutrophil recruitment in human blood, extracellular histones specifically did not directly act on neutrophils, rather they targeted monocytes to induce chemokine production [24].

Under healthy conditions, neutrophils within the vasculature do not cause damage even when they are primed by exposure to inflammatory mediators. The healthy pulmonary endothelium instructs the primed cells to ‘de-prime’ and return back to a quiescent state within the vasculature [25]. It is only when neutrophils confront activated markers on the pulmonary endothelium such as P-selectin, ICAM-1, etc., will they adhere to the endothelium and become activated [26]. Additionally, abnormal activation of the coagulation cascade contributes to lung injury, and amplifies pulmonary inflammation by inducing disturbances in microcirculation and increasing endothelial permeability [27].

The lung endothelial cell surface is the common platform for orchestrating inflammation and coagulation cascade activation [28]. Once challenged by inflammatory mediators, the lung endothelium can shift from their normal anti-thrombotic and anti-inflammatory phenotype to pro-thrombotic and pro-adhesive properties [29]. P-selectin and vWF are stored within WPBs of endothelium. In response to abnormal challenges, P-selectin can be rapidly translocated to cell surface and vWF can be released through exocytosis, both of which are involved with endothelial activation. The translocated P-selectin can mediate leukocyte tethering and rolling on activated endothelial cells [30]. In addition to being a highly pro-thrombotic protein, vWF is also an important pro-inflammatory mediator, contributing to neutrophil diapedesis via modulating the integrity of the endothelial barrier [31–33]. Additionally, released vWF can directly interact with DNA from neutrophil extracellular traps to aggravate inflammatory damage [34]. This concept highlights the importance of understanding the mechanisms by which inflammatory mediators interact with endothelium.

The doses of exogenous histones used during in vivo and in vitro experiments were different from the levels of histone H4 observed in LPS-challenged mice. The following facts maybe show some explanation. Many serious diseases can act as the first hit to cause injury and the uncontrolled inflammation is just the second hit to aggravate the damage [5, 6]. When histones were used as the sole etiology to investigate the injury mechanism in the experiment, they must cause both the first and the second hit, thus the doses of exogenous histones used were much higher.

Surely during the metabolic process histones need several posttranslational modifications including acetylation, methylation, glycosylation, ubiquitination, and deimination [35–37]. Extracellular histones may also undergo some modifications to change their inflammatory characteristic, which need to be further investigated.

Extracellular histones in circulation are lung-targeted damage mediators, which can bind pulmonary capillary endothelium with priority. This is consistent with the phenomenon that the lung is the most vulnerable organ in systemic inflammatory damage.

## Conclusions

In conclusion, extracellular histones are pro-inflammatory mediators in LPS-induced ARDS in mice. As far as our experiment results are concerned, extracellular histone-induced endothelial activation promotes neutrophil adhesion and subsequent activation though extracellular histones have little direct effects on neutrophil. Thus, endothelial activation is important for extracellular histone-induced inflammatory injury.

## Abbreviations

anti-H4: anti-histone H4 antibody
ARDS: Acute respiratory distress syndrome
CTH: Calf thymus histones
DAMP: Damage-associated molecular pattern
EGM-2: Endothelial growth medium-2
HBSS: Hanks’ balanced salt solution
LPS: Lipopolysaccharide
MLVEC: Mouse lung vascular endothelial cell
MOF: Multiple organ failure
MPO: Myeloperoxidase
PAMP: Pathogen-associated molecular pattern
PSGL-1: P-selectin-glycoproteinligand-1
sTM: soluble thrombomodulin
TLR: Toll like receptor
vWF: von Willebrand factor
WPB: Weibel-Palade body
